# Comprehensive Evaluation of the Genotoxic Potential of Food Additive Titanium Dioxide in Human Intestinal Cell Systems

**DOI:** 10.3390/ijms262412026

**Published:** 2025-12-14

**Authors:** Han-Na Nam, Su-Min Jeong, Su-Bin Kim, Soo-Jin Choi

**Affiliations:** Department of Food Science and Technology, Seoul Women’s University, Seoul 01797, Republic of Korea; nhn0318@swu.ac.kr (H.-N.N.); zzzin_pang@swu.ac.kr (S.-M.J.); qlsqlskim@swu.ac.kr (S.-B.K.)

**Keywords:** titanium dioxide, food additives, genotoxicity, oxidative stress, DNA damage, chromosomal aberration, micronucleus

## Abstract

Titanium dioxide (TiO_2_), widely recognized as a whitening food additive, has been extensively employed in food products such as confectionery, sauces, and coffee creamers. The potential genotoxicity of TiO_2_ has recently raised increasing concern, especially after the European Union prohibited its use as a food additive due to genotoxicity risks. The contradictory outcomes of in vitro and in vivo studies emphasize the necessity for more rigorous and systematic evaluation. In this study, we assessed the potential genotoxicity of food-grade TiO_2_ in human intestinal cell lines and intestinal barrier models. Three distinct genotoxicity assays were conducted: the comet assay (DNA tail formation), chromosomal aberration analysis, and the micronucleus assay. The results revealed that TiO_2_ exposure led to DNA damage primarily associated with oxidative stress in various intestinal cell systems at actual intake levels, regardless of metabolic activation; however, it did not trigger chromosomal aberrations or micronucleus formation. Thus, TiO_2_ appears to cause in vitro genotoxic damage at the DNA level, but not at macroscopic endpoints, such as chromosomal aberrations or micronucleus formation. Further in-depth in vivo study is required to definitively determine the potential genotoxicity of TiO_2_ in the food industry.

## 1. Introduction

The food additive titanium dioxide (TiO_2_) is used as a whitening and brightening agent in the food industry and is applied in candies, chewing gums, baked goods, coffee creamers, chocolates, dairy products, sauces, and dressings. It is a naturally occurring, inorganic, and insoluble pigment and can be synthesized using physical, chemical, and biological approaches [1,2]. TiO_2_ has been used as a food additive for over five decades—it was approved by the United States Food and Drug Administration (FDA) as a food additive in 1966, with the condition that its content in food not exceed 1% of the weight [3]. In 2008, the European Union (EU) authorized TiO_2_ as a food additive, listed as E171 under Regulation (EC) No. 1333/2008 [4].

The EU banned TiO_2_ (E171) as a food additive in 2022, based on findings of the European Food Safety Authority (EFSA). In 2021, EFSA re-evaluated the safety aspect of TiO_2_ and concluded that E171 can no longer be considered safe when used as a food additive due to a genotoxic concern [5]. Indeed, the expert Panel on Food Additives and Flavorings of EFSA did not exclude potential genotoxicity effects of TiO_2_, which might cause DNA breaks and chromosomal damage, but not gene mutations [5]. The Panel noted that TiO_2_ particles can accumulate in the body, despite their low oral absorption, and that the currently available data did not provide definitive evidence of general toxicity [5]. The re-evaluation of TiO_2_ was initiated by the study, which showed that food-grade TiO_2_ at 10 mg/kg/day via drinking water for 100 days promoted colon microinflammation, preneoplastic lesions, and aberrant crypt development in the rat colon [6]. Studies have also demonstrated in vitro DNA oxidation and homeostasis alteration in human intestinal epithelial cells due to E171 exposure [7,8,9].

In contrast, the Joint Food and Agriculture Organization (FAO) of the United Nations/World Health Organization (WHO) Expert Committee on Food Additives (JECFA) re-evaluated the safety of TiO_2_ in November 2023 and concluded that it is safe based on poor absorption from the gastrointestinal tract, very low oral bioavailability, no evidence for carcinogenic, reproductive, or developmental toxicity, and limited available evidence for genotoxicity [10]. Considering the very low oral absorption of TiO_2_ and the absence of any identified hazard associated with TiO_2_ in the diet, JECFA reaffirmed its acceptable daily intake as “not specified”, established in 1969 [11]. The FDA did not raise concern related to the potential genotoxicity of TiO_2_ according to the data available, and remarked that orally exposed TiO_2_ was not carcinogenic in mice or rats in carcinogenicity studies of the National Toxicology Program [12]. The United Kingdom’s Food Standards Authority, Health Canada, and Food Standards Australia New Zealand conclude that there is currently no evidence to suggest that dietary exposure to food-grade TiO_2_ is a concern for human health [13,14,15].

Indeed, no evidence of aberrant crypt foci, a potential precursor to colorectal cancer, was observed following administration of E171 to rats via the diet at 267 mg/kg/day for 100 days [16]. A Laboratory Pharmacology and Toxicology report performed a one-generation reproductive toxicity study of E171 in rats via the diet at doses up to 1000 mg/kg/day, but no aberrant crypt foci were observed [5]. Currently, in vivo genotoxicity studies with food-grade TiO_2_ administered via the diet are not sufficiently available. Moreover, the administration route via diet, drinking water, or oral gavage can be an important factor affecting the toxicity of TiO_2_. Indeed, our recent report demonstrated that the interactions between TiO_2_ and food (albumin) or bio-matrix (fetal bovine serum, FBS) can reduce DNA damage to non-treated control levels in human intestinal epithelial Caco-2 cells, attributed to the increased hydrodynamic diameters resulting from the interactions [17]. We also showed that the TiO_2_-induced DNA damage was associated with reactive oxygen species (ROS) generation and oxidative stress [17].

The aim of this study was to provide a systematic evaluation of the potential genotoxicity of TiO_2_ particles of two different sizes (T3 and T4). We employed human intestinal cell systems, including the Caco-2 and HT-29 cell lines, as well as intestinal barrier models such as Caco-2 monolayer, Caco-2/HT-29 co-culture, and follicle-associated epithelium (FAE). The assessment focused on multiple genotoxicity-related endpoints, including oxidative stress, DNA damage, chromosomal aberrations, and micronuclei formation.

## 2. Results and Discussion

### 2.1. Characterization of TiO_2_

**Figure 1:**
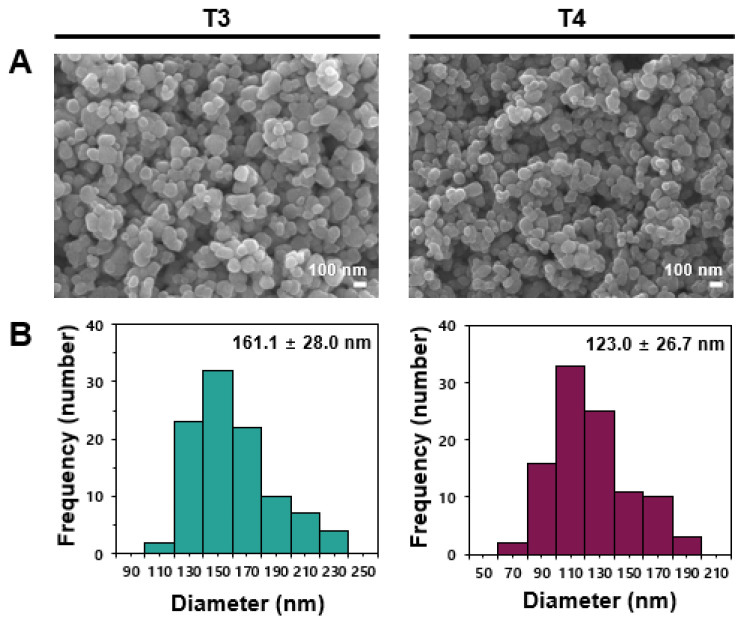
(**A**) Scanning electron microscopic (SEM) images and (**B**) constituent particle size distribution of food-grade TiO_2_ particles (T3 and T4). Particle size distribution was analyzed by measuring 100 randomly chosen particles from SEM micrographs.

Based on our previous report, two different sizes of TiO_2_ particles (T3 and T4) were selected to represent the largest and smallest particles, respectively, among the five commercially available food-grade TiO_2_ particles examined [18]. Scanning electron microscopy (SEM) analysis showed that average constituent particle sizes of T3 and T4 were 161.1 ± 28.0 nm and 123.0 ± 26.7 nm, respectively (Figure 1), which do not fall within the nanoparticle (NP) size range of 1 to 100 nm. A small portion (18%) of NP below 100 nm was found for T4, but not for T3 (Table 1), indicating the presence of NPs in some cases. The hydrodynamic diameters of T3 and T4 in distilled and deionized water (DDW) were 304.7 ± 0.9 nm and 295.7 ± 2.9 nm, respectively, suggesting an aggregate fate in aqueous solution. Both particles had an anatase crystal structure and extremely low solubility (<0.2%) even in the presence of matrices, as determined in our previous reports [17,18].

### 2.2. Cytotoxicity and ROS Generation of TiO_2_

**Figure 2:**
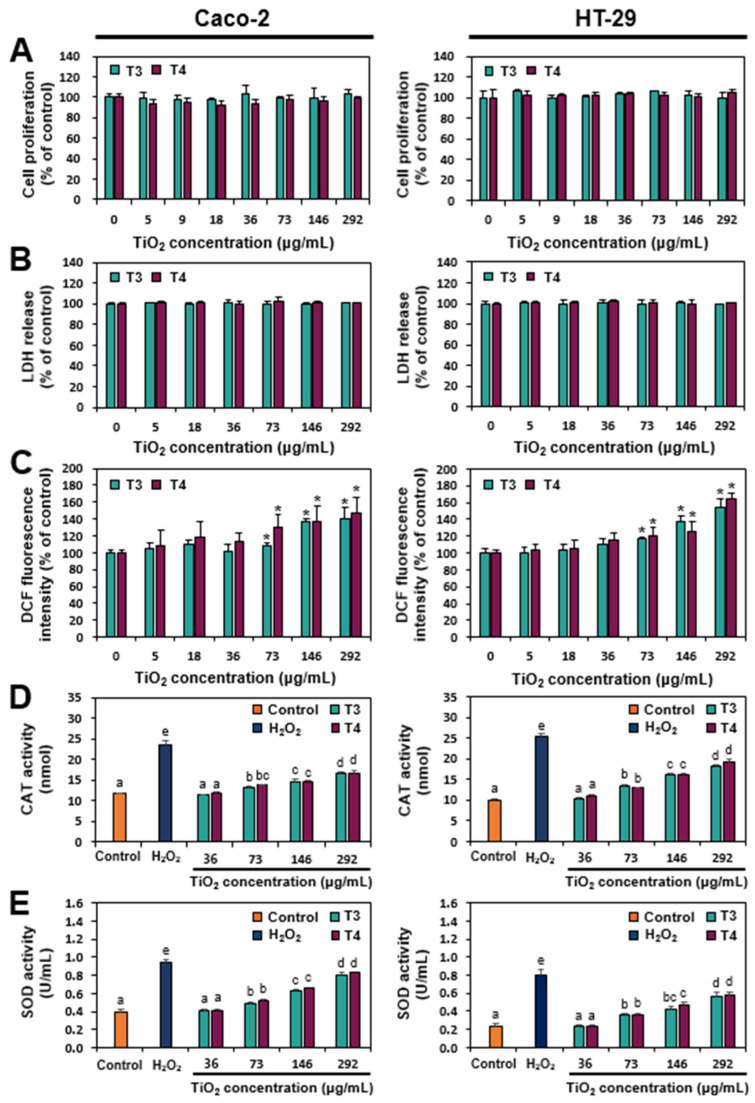
(**A**) Cell proliferation, (**B**) lactate dehydrogenase (LDH) leakage, (**C**) reactive oxygen species (ROS) production, and (**D**,**E**) antioxidant enzyme (catalase, CAT; superoxide dismutase, SOD) activities in Caco-2 and HT-29 cells treated with TiO_2_ particles (T3 and T4) for 24 h. * indicates statistically significant differences in comparison with untreated control cells (*p* < 0.05). Different lowercase letters (a, b, c, d, e) above the bars represent significant differences among treated groups (untreated control, H_2_O_2_, different concentrations of TiO_2_) (*p* < 0.05). Abbreviation: DCF, dichlorofluorescein.

The cytotoxicity of two different food-grade TiO_2_ particles was evaluated in terms of cell proliferation inhibition, lactate dehydrogenase (LDH) release, and ROS generation in two different human intestinal Caco-2 and HT-29 cells. The highest exposure concentration was set at 292 μg/mL considering maximum usage levels in commercial products from the Manufacturing Report (2018–2019) and daily intake levels from the Item and National Food and Nutrition Statistics (2017) in the Republic of Korea [19]. The volume of human intestinal fluid (100 mL) was also taken into consideration for the cell culture experiments [20], which had been used in our previous report [17]. Figure 2 shows that both T3 and T4 particles did not inhibit the cell proliferation of Caco-2 and HT-29 cells (Figure 2A), and moreover, no significant cell death determined by LDH release was found (Figure 2B). In contrast, T3 and T4 particles induced ROS generation at above 73 μg/mL in a concentration-dependent manner, without significant differences between the two particles (Figure 2C) (*p* > 0.05). To confirm oxidative stress caused by two TiO_2_ particles, the activities of antioxidant enzymes, catalase (CAT) and superoxide dismutase (SOD), were evaluated following particle exposure. The results demonstrate that the antioxidant enzyme activities increased in a concentration-dependent manner in both Caco-2 and HT-29 cells exposed to T3 and T4 particles (Figure 2D,E). These results clearly indicate that food-grade TiO_2_ particles did not affect cell proliferation or death but induced oxidative stress regardless of particle size at actual intake levels. It was reported that TiO_2_ particles cause oxidative stress in cell lines, which seems to be related to their photocatalytic activity, generating free radicals and ROS [21,22,23].

### 2.3. DNA Damage Caused by TiO_2_ in Human Intestinal Cell Lines

**Figure 3:**
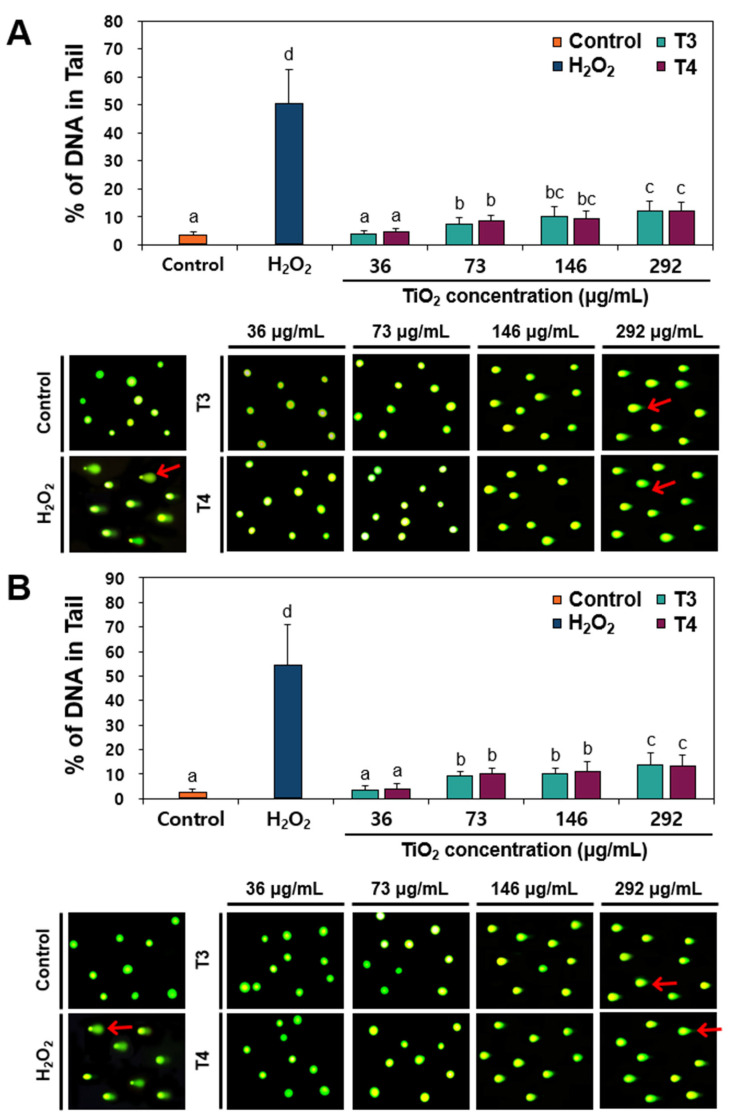
DNA fragmentation in (**A**) Caco-2 and (**B**) HT-29 cells exposed to TiO_2_ particles (T3 and T4) for 24 h, as determined by comet assay. Images were magnified at 20×. DNA strand breaks were analyzed and expressed as % of DNA in the tail. H_2_O_2_ (100 μM) was used as a positive control. Red arrows indicate DNA in the comet tails. Different lowercase letters (a, b, c, d) above the bars represent significant differences among treated groups (untreated control, H_2_O_2_, different concentrations of TiO_2_) (*p* < 0.05).

DNA damage in two different human intestinal cells exposed to food-grade TiO_2_ particles was evaluated using the comet assay as an indicator of potential genotoxicity. The assay measures the migration of broken DNA fragments out of a cell’s nucleus under an electric field. Thus, the proportion of DNA in the comet tail provides a quantitative measure of DNA fragmentation. The results demonstrate that both T3 and T4 particles caused a concentration-dependent increase in DNA tail formation at concentrations above 73 μg/mL (Figure 3) in both Caco-2 (Figure 3A) and HT-29 (Figure 3B) cells, aligning with observed ROS generation (Figure 2C) and antioxidant activity (Figure 2D,E). It is worth noting that the increased percentages of DNA in tails caused by TiO_2_ exposure were about 1/4-fold less than the DNA damage caused by a positive control, H_2_O_2_ exposure. These findings indicate that TiO_2_ induced DNA damage, although the damage levels were low. Several studies have reported that TiO_2_ NPs induce DNA damage in cell lines, as evidenced by the comet assay [24,25,26], which is consistent with our results. However, conflicting results have also been reported, demonstrating negative responses to TiO_2_ exposure [27,28]. The types of TiO_2_ materials tested (food- or general-grade), particle size distribution, concentration exposed, or cell line systems may lead to different results.

### 2.4. Effect of a Metabolic Activation on DNA Damage Caused by TiO_2_

**Figure 4:**
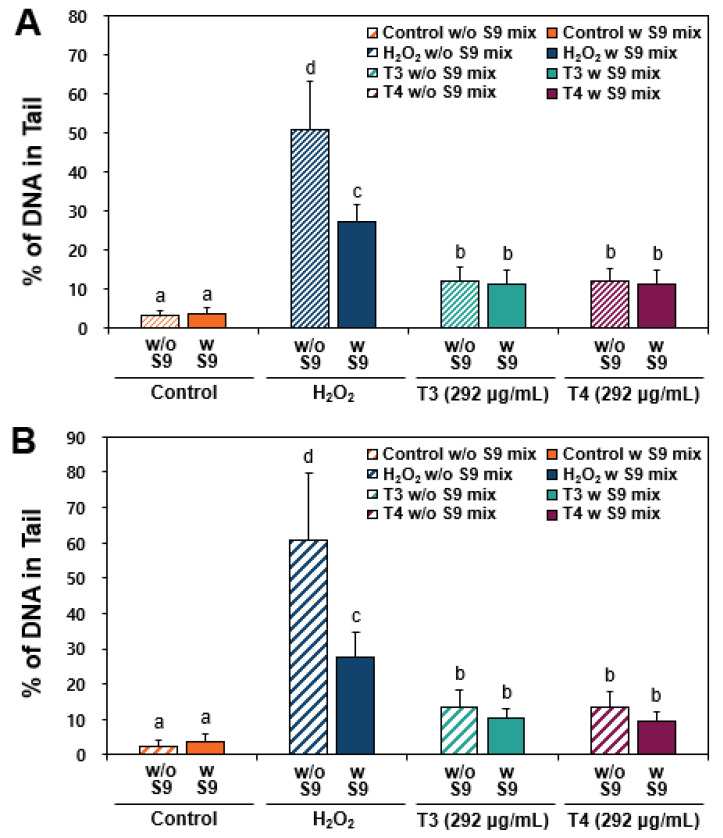
DNA fragmentation in (**A**) Caco-2 and (**B**) HT-29 cells exposed to TiO_2_ particles (T3 and T4) for 24 h, as determined by comet assay with (w) or without (w/o) an S9 mix. Images were magnified at 20×. DNA strand breaks were analyzed and expressed as % of DNA in the tail. H_2_O_2_ (100 μM) was used as a positive control. Different lowercase letters (a, b, c, d) above the bars represent significant differences among treated groups (untreated control, H_2_O_2_, 292 μg/mL of T3, and 292 μg/mL of T4) (*p* < 0.05). No significant differences were found between groups with and without S9 mix (*p* > 0.05).

The effect of metabolic activation on DNA damage was assessed by examining TiO_2_-induced DNA tail formation in the presence of an S9 mix. The S9 fraction is a liver extract that contains active liver enzymes, stimulating hepatic metabolism. Indeed, certain substances require metabolic conversion to exert toxicity or mutagenicity, or to undergo detoxification. Figure 4 shows that increased DNA tail levels by both T3 and T4 exposure were not significantly affected by the presence of S9 mix (*p* > 0.05), whereas H_2_O_2_-induced DNA tails decreased by metabolic activation in two different human intestinal cells. These results suggest that TiO_2_ itself can directly cause DNA damage, presumably via its ROS generation characteristics [17,29], and that such damage was not reversed by S9-mediated metabolic activation. In contrast, the decrease in H_2_O_2_-induced DNA tail formation in the presence of S9 mix indicates that its toxic effects may be mitigated via metabolic activation [30]. To the best of our knowledge, the effect of metabolic activation on DNA damage caused by TiO_2_ was not well demonstrated, indicating the scientific significance of this result.

### 2.5. DNA Damage Caused by TiO_2_ in Human Intestinal Barrier Models

**Figure 5:**
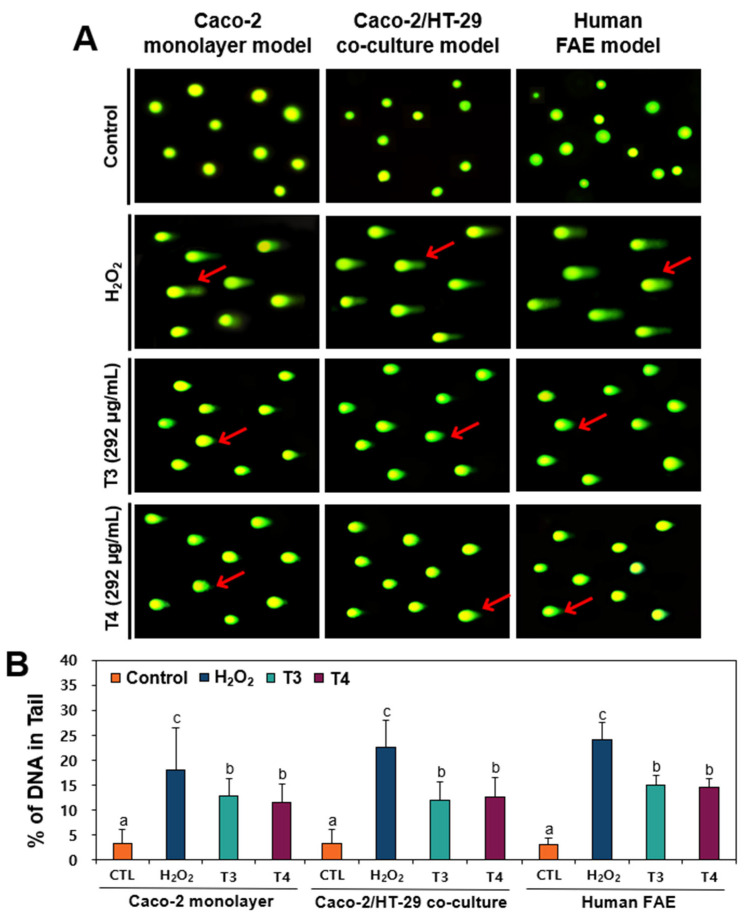
(**A**) Representative images and (**B**) percentages of DNA fragmentation in three different human intestinal barrier models exposed to TiO_2_ particles (T3 and T4) for 24 h, as determined by comet assay. Images were magnified at 20×. DNA strand breaks analyzed and expressed as % of DNA in tail. H_2_O_2_ (100 μM) was used as a positive control. Red arrows indicate DNA in the comet tails. Different lowercase letters (a, b, c) above the bars represent significant differences among treated groups (untreated control, H_2_O_2_, 292 μg/mL of T3, and 292 μg/mL of T4) (*p* < 0.05).

DNA tail formation caused by TiO_2_ particles as an indicator of DNA damage was further assessed in three different human intestinal barrier models: 2D Caco-2 monolayer, 2D Caco-2/HT-29 co-culture, and 3D human FAE models. It has been reported that toxicity results can be influenced by different biological test systems such as cultured cell lines and 2D/3D models [31,32]. Nevertheless, most studies evaluated the potential genotoxicity of TiO_2_ particles in cell lines [33]; however, diverse intestinal barrier models were not used. Hereafter, the experiments were performed only without S9 mix because no effect of metabolic activation on TiO_2_-induced DNA damage was found (Figure 4). The results demonstrate that significantly increased percentages of DNA in the comet tails were found in three human intestinal barrier models exposed to both T3 and T4 particles (*p* < 0.05), without significant differences between the two particles (*p* > 0.05) (Figure 5). However, DNA damage levels were significantly lower than those observed under H_2_O_2_ exposure. These findings are highly consistent with the comet assay results in two different human intestinal cell lines (Figure 3), suggesting the in vitro genotoxic potential of TiO_2_ particles. It is worth noting that our previous report demonstrated in vitro intestinal transport (below 0.13%) of T3 and T4 particles through the human intestinal barriers, as well as their ex vivo intestinal absorption (below 1.5%), using an everted gut sac model [17]. This supports the genotoxic concerns regarding TiO_2_ particles, despite low intestinal transport and absorption.

### 2.6. 8-Hydroxy-2′-Deoxyguanosine (8-OHdG) Generation Caused by TiO_2_

**Figure 6:**
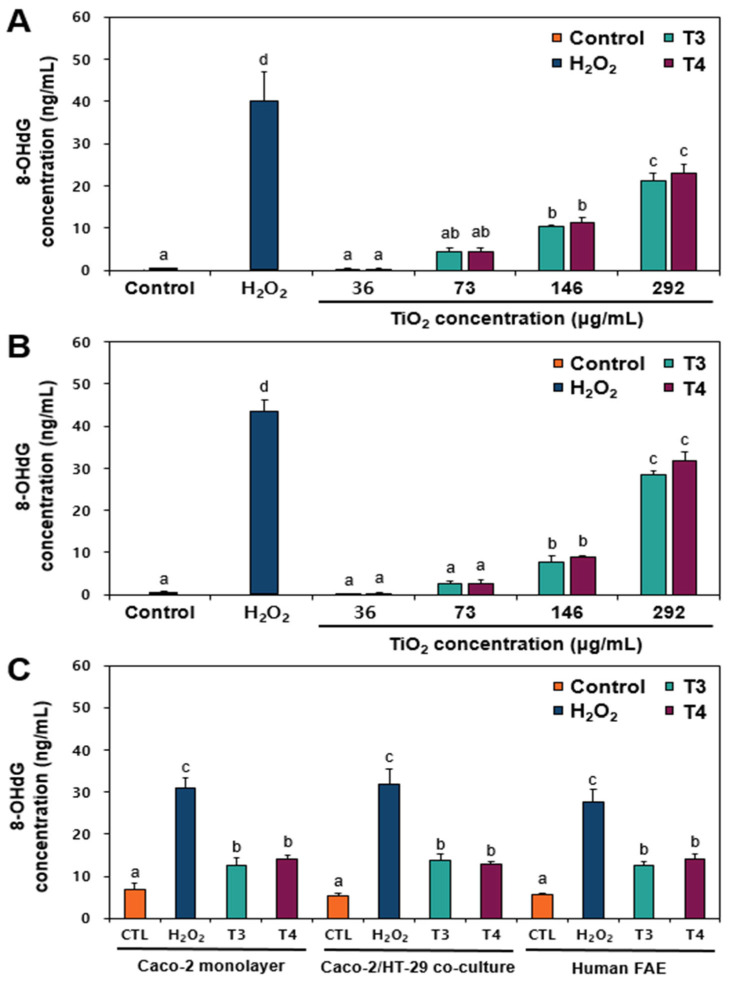
8-Hydroxy-2′-deoxyguanosine (8-OHdG) levels in (**A**) Caco-2 cells, (**B**) HT-29 cells, and (**C**) human intestinal barrier models exposed to TiO_2_ particles (T3 and T4) for 24 h. Control represents basal 8-OHdG levels of untreated cells without particles. H_2_O_2_ (100 μM) was used as a positive control. Different lowercase letters (a, b, c, d) above bars denote significant differences among treated groups (untreated control, H_2_O_2_, and different concentrations of TiO_2_, and 292 μg/mL of T3 or T4) (*p* < 0.05). Abbreviations: CTL, control; FAE, follicle-associated epithelium.

The relationship between DNA damage and oxidative stress was assessed by measuring intracellular 8-OHdG levels in both human intestinal cell lines and barrier models. 8-OHdG, an oxidized derivative of deoxyguanosine, represents one of the predominant products of DNA oxidation. Accordingly, its intracellular concentration serves as a biomarker for oxidative stress-related DNA damage. Figure 6 shows that intracellular 8-OHdG levels significantly increased to above 73 μg/mL in a concentration-dependent manner (*p* < 0.05), regardless of particle size (T3 and T4) (*p* > 0.05), in Caco-2 and HT-29 cell lines (Figure 6A,B). Significantly increased 8-OHdG concentrations were also found in the Caco-2 monolayer model, Caco-2/HT-29 barrier model, and FAE model (Figure 6C). These findings clearly suggest that DNA damages caused by food-grade TiO_2_ particles are closely associated with ROS generation and oxidative stress. Indeed, our previous study demonstrated that oxidative stress-related DNA fragmentation caused by food-grade TiO_2_ could be mitigated through its interaction with bio- or food matrices, such as FBS and albumin [17]. In vitro genotoxicity studies of TiO_2_ particles have also shown that TiO_2_-induced genotoxicity occurs mainly through ROS-mediated oxidative stress [34]. The mechanisms underlying oxidative stress-related DNA strand breaks induced by TiO_2_ particles must be elucidated, including mitochondrial dysfunction and imbalance in antioxidant defenses.

### 2.7. In Vitro Chromosomal Aberration Caused by TiO_2_

Chromosomal abnormality refers to any change in the structure or number of chromosomal DNA. Numerical aberration involves aneuploidy (abnormal number of chromosomes) and polyploidy (more than two paired sets of chromosomes). Structural aberration includes break, deletion, fragment, and exchange. The comet assay directly measures DNA fragmentation, such as single- and double-strand breaks in individual cells, while chromosomal aberrations are large-scale structural changes to entire chromosomes or chromatids, including deletions or rearrangements. Thus, chromosomal aberration assays detect a more visible and macroscopic consequence of DNA damage and are used to complement the comet results. We evaluated chromosomal aberration in Caco-2 cells because the different human intestinal cell systems used in this study showed the same tendency (Figure 3, Figure 4, Figure 5 and Figure 6). Table 2 shows that the positive controls mitomycin C (MMC) and cyclophosphamide (CPA), used for the absence and presence of S9 mix, induced 15.5% and 16.5% structural aberrations, respectively, indicating consistent results with previous studies [35,36]. The cells exposed to T3 and T4 showed a small frequency of structural aberrations (2–3%) as observed in non-treated control cells, including break, exchange, and fragmentation. It is worth noting that gaps were recorded, but not included in the total aberration frequency according to OECD test guideline 473 [37]. These results suggest that two different food-grade TiO_2_ particles did not induce chromosomal aberrations in Caco-2 cells. Patel et al. reported that TiO_2_ NPs with an average particle size of 20.3 nm obtained from Sigma-Aldrich showed a significant increase in chromosome aberrations to above 75 μM in human peripheral blood lymphocytes [38]. In contrast, the in vitro genotoxicity study of TiO_2_ (Green Sludge Titanium) reported no statistically significant positive frequency in chromosomal aberrations in a Chinese hamster lung cell line [35]. Food additive TiO_2_ E171 was reported to induce chromosomal aberration [39], but conflicting results were also found [40]. Particle size, material type or biosystems tested, method prepared, and data quality seem to critically lead to inconsistent results [41].

### 2.8. In Vitro Micronucleus Formation Caused by TiO_2_

Micronuclei are small nuclear entities produced when chromosomes or fragments fail to integrate into the daughter nuclei during cell division, reflecting genotoxic damage and chromosomal instability. A DNA tail, observed in the comet assay, is a distinctive structure formed by fragmented DNA strands, whereas extra-nuclear micronuclei are small bodies that contain chromosome fragments or entire chromosomes resulting from chromosomal aberrations or DNA damage. A micronucleus formation assay was further performed to confirm that food-grade TiO_2_ particles had no effect on in vitro chromosome aberrations obtained in this study. Indeed, DNA damage observed in the comet assay could contribute to chromosome fragments. Table 3 shows that for the absence and presence of S9 mix, respectively, the positive controls methyl methanesulfonate (MMS) and CPA induced about 9% frequency of micronucleated binucleated cells (MNBNs), as reported in other studies [42,43]. Meanwhile, Caco-2 cells exposed to food-grade TiO_2_ particles showed a similar amount of MNBNs (2.5–3%) compared to untreated control cells (2.5–3%), indicating no effect on micronucleus formation (Table 3). These results were highly consistent with in vitro chromosomal aberration results (Table 2). Contradictory results were reported, showing that three different TiO_2_ particles (food additive E171, NPs, and micro-sized particles) induced ROS formation, DNA breaks, and chromosome damage in human intestinal cells [44]. In contrast, similarly to the present study, no effect of TiO_2_ (E171) on micronuclei formation has been demonstrated [45,46]. It is important to note that DNA strand breaks were not observed after repeated intragastric administration of food-grade TiO_2_ (E171) for 15 days in rat liver cells [47]. Commercial food additive TiO_2_ did not induce DNA fragmentation in rats, as determined by the in vivo comet assay following 15 days of consecutive intragastric administration [46].

The present study demonstrated that commercially available TiO_2_ particles induced DNA fragments, but not chromosome aberration or micronuclei formation at actual intake levels. It is likely that TiO_2_ particles could cause DNA strand breaks associated with oxidative stress, but the extent of DNA damage appears to be insufficient to trigger chromosomal aberrations. As noted by Werheit [48], it is not appropriate to compare the toxicity results of well-dispersed NPs with those of TiO_2_ particles present in food. Interactions between TiO_2_ particles and food matrices surely occur, which can reduce the genotoxic potential of TiO_2_ particles [17]. Moreover, in vivo oral absorption of TiO_2_ particles is extremely low [49,50], which may also explain their lack of genotoxic effects at actual dietary intake levels.

## 3. Materials and Methods

### 3.1. Food-Grade TiO_2_ and Characterization

Commercially available representative large (T3)- and small (T4)-sized food-grade E171 TiO_2_ particles were identified in our previous report and used in this study [17,18]. The particles (1 mg/mL) were dispersed in DDW, followed by stirring for 30 min and sonicating for 5 min (160 Watts, Bransonic 580, Branson Ultrasonics, Danbury, CT, USA) just before all experiments.

Field emission–scanning electron microscopy (SEM, JSM-7100F, JOEL, Tokyo, Japan), dynamic light scattering with a Zetasizer Nano System (Malvern Instruments, Worcestershire, UK), and Ni-filtered CuKα radiation (D2phaser, Bruker AXS Inc., Madison, WI, USA) were used to determine constituent particle sizes, hydrodynamic radii, and crystalline phases of TiO_2_ particles, respectively.

### 3.2. Cell Culture and Cytotoxicity

Human intestinal epithelial Caco-2 cells and HT-29 cells were purchased from Korean Cell Line Bank (Seoul, Republic of Korea) and cultured in complete minimum essential medium and Roswell Park Memorial Institute 1640 medium, respectively, containing heat-inactivated FBS (10%), penicillin (100 units/mL), and streptomycin (100 μg/mL) under 5% CO_2_ atmosphere at 37 °C.

Cell proliferation inhibition and membrane damage caused by TiO_2_ particles were evaluated with water-soluble tetrazolium salt-1 (WST-1; Roche, Molecular Biochemicals, Manheim, Germany) and a CytoTox 96 Non-Radioactive Cytotoxicity assay (Promega, Madison, WI, USA), respectively. For the WST-1 assay, cells (1 × 10^4^ cells/100 μL) were exposed to designated concentrations of TiO_2_ for 24 h, followed by further incubation for 4 h after treatment with WST-1 solution (10 μL), and finally, absorbance at 440 nm was performed with a microplate reader (Infinite^®^ M Plex, Tecan, Männedorf, Switzerland). LDH release as a marker of membrane damage was assessed by treating cells (4 × 10^4^ cells/mL) with TiO_2_. After 24 h, 50 μL of the cell culture medium was reacted with the same volume of substrate solution for 30 min at room temperature, and 50 μL of stop solution was added. Absorbance values at 492 nm were then obtained using a microplate reader (Infinite^®^ M Plex, Tecan). The highest concentration of 292 μg/mL was calculated as follows [17,20]: maximum usage level (%) × daily intake (g)/volume of intestinal fluids (100 mL).

### 3.3. ROS Generation and Antioxidant Enzyme Activity

Intracellular ROS levels were measured with a peroxide-sensitive fluorescent probe, 2′,7′-dichlorofluorescein diacetate (H_2_DCFDA; Molecular Probes Inc., Eugene, OR, USA). Cells (1 × 10^4^ cells/100 μL) were exposed to designated concentrations of TiO_2_ for 24 h. Then, the cells were incubated with H_2_DCFDA for an additional 30 min at 37 °C in the dark. After washing with phosphate-buffered saline (PBS), the levels of intracellular dichlorofluorescein fluorescence were determined using a microplate reader (Infinite^®^ M Plex, Tecan) with excitation and emission wavelengths set at 485 nm and 530 nm, respectively.

The activities of the antioxidant enzymes CAT and SOD, as markers of oxidative stress, were evaluated in cells (1 × 10^6^ cells/2 mL) exposed to a designated concentration of TiO_2_ for 24 h. This evaluation was performed using CAT and SOD assay kits from Cayman Chemical Co. (Ann Arbor, MI, USA), in accordance with the provided protocols.

### 3.4. In Vitro Intestinal Barrier Models

Two different models of the human intestinal epithelial tight junction barrier were established. A Caco-2 monoculture model was prepared by incubating Caco-2 cells (4.5 × 10^5^ cells/well) on apical inserts for 21 days (transepithelial electrical resistance; TEER values ≥ 300 Ω cm^2^) [51]. A Caco-2/HT-29 co-culture model (4.5 × 10^5^ cells/well) was prepared by culturing Caco-2:HT-29 cells at a ratio of 9:1 for 21 days (TEER values ≥ 350 Ω cm^2^) [52].

An FAE model, representing microfold cells in Peyer’s patches, was prepared by culturing Caco-2 cells (1 × 10^6^ cells/well) on apical insert sides for 14 days, followed by further culturing Raji B cells (1 × 10^6^ cells/well) prepared in Dulbecco’s modified eagle’s medium in basolateral insert parts for 5 days (TEER values ranged from 150 Ω cm^2^ to 200 Ω cm^2^). The medium containing TiO_2_ (292 μg/mL) was placed on apical inserts, and incubation for 24 h was carried out.

### 3.5. Comet Assay

Cells (1 × 10^6^ cells/2 mL) or intestinal barrier models were incubated with TiO_2_ (292 μg/mL) for 24 h in the absence of S9 mix. To assess the effect of metabolic activation, cells were exposed to TiO_2_ (292 μg/mL) for 4 h in the presence of S9 mix, followed by a recovery period of 20 h in complete medium. The S9 mix was prepared at a final concentration of 1.5% by combining S9 fraction (rat liver S9 homogenate in KCl; MP Biomedicals, Irvine, CA, USA) with a cofactor solution (Cofactor III; Genogen Co., Ltd., Cheongju, Republic of Korea) at a ratio of 2:4.7. H_2_O_2_ (100 μM) was used as a positive control. After treatment, the cells were incubated with 5 mM ethylene diamine tetraacetic acid (1 mL) for 40 s, washed with PBS, detached with a scraper, centrifuged, and re-suspended (1 × 10^5^ cells/1 mL) in ice-cold PBS.

A comet assay was conducted with a kit obtained from R&D systems (Minneapolis, MN, USA), following the instructions provided by the manufacturer. The cell suspensions were combined with low-melting point agarose at a 1:10 (*v*/*v*) ratio, and 75 μL of the mixture was promptly placed onto the comet slide. The slides were kept at 4 °C in the dark for 30 min to allow gelation, followed by immersion in a lysis solution maintained at 4 °C for 1 h. After removal of the lysis solution, the slides were incubated in alkaline solution (pH > 13) for 30 min in the dark at room temperature. The alkaline solution was removed, and electrophoresis was carried out at 20 V and 300 mÅ for 40 min at 4 °C under dark conditions. After washing with DDW, the slides were immersed in 70% ethanol for 5 min, allowed to air-dry, and then stained with 100 μL of Cygreen dye for 30 min in the dark at 4 °C. Analysis was performed with an Axioskop 2 plus fluorescent microscope (Carl Zeiss, Oberkochen, Germany) and Cometscore 2.0 imaging software. Comet tail lengths were measured, and the percentage (%) of DNA in the tail (the fraction of DNA in the tail divided by the total amount of DNA) was determined, with at least 200 cells analyzed per sample.

### 3.6. 8-OHdG Assay

Cells (1 × 10^6^ cells/2 mL) were treated with designated concentrations of TiO_2_ for 24 h. A high concentration of 292 μg/mL TiO_2_ was used for intestinal barrier models. H_2_O_2_ (100 μM) was used as a positive control. The DNA was extracted using the AccuPrep^®^ Genomic DNA Extraction kit (Bioneer, Daejeon, Republic of Korea) following the instructions provided by the manufacturer. A total of 20 μg of extracted DNA (A260/A280 ≥ 2) in each sample was denatured at 95 °C for 5 min, followed by rapid cooling on ice. The denatured DNA was then treated with 0.1 μL of nuclease P1 and incubated at 50 °C for 1 h in 20 mM sodium acetate (pH 5.3) to facilitate nucleoside formation. Alkaline phosphatase was added to the mixture and incubated at 37 °C for 1 h to achieve phosphate decomposition. The pH was then adjusted to 7.5–8.5, and the solution was heated to boiling for 10 min. The prepared samples were kept on ice until analysis. Quantification of 8-OHdG, a nucleoside derived from the oxidative modification of guanosine, was performed using the 8-OHdG enzyme-linked immunosorbent assay kit (Abcam, Cambridge, UK) in accordance with the manufacturer’s instructions.

### 3.7. In Vitro Chromosomal Aberration Assay

Caco-2 cells (1.5 × 10^5^ cells/2 mL) were treated with TiO_2_ (292 μg/mL) for 24 h without S9 mix (positive control: 0.2 μg/mL MMC) or incubated with particles for 4 h followed by a recovery time for 20 h in the presence of S9 mix (positive control: 10 μg/mL CPA). After removing the supernatant and washing three times with PBS, the cells were further incubated for 30 h (1.5 cell cycles) in complete medium. Colcemid solution was treated for 2 h just before the 30 h treatment to arrest cell division at the metaphase state of mitosis. The cell pellets were obtained by washing with PBS and centrifugation (365× *g*, 5 min), re-suspended in 3 mL of 0.075 M KCl solution, and incubated at 37 °C for 30 min. Subsequently, 2 mL of fixative solution (methanol–acetic acid = 3:1, 4 °C) was added to the cell suspension and centrifuged (365× *g*, 5 min). After removing the supernatant, 5 mL of fixative solution was added, re-suspended, and centrifuged (730× *g*, 5 min). This procedure was repeated twice. The cell pellets were re-suspended in 1 mL of fixative solution, and 100 μL of the cell suspension was dropped onto a slide and air-dried. The slides were then stained with 5% Giemsa solution for 20 min, rinsed with distilled water, dried, and examined under a microscope (Axioskop 2 plus, Carl Zeiss). At least 200 well-spread metaphases were examined, and numerical (aneuploid, polyploid) and structural (break, deletion, fragment, exchange) aberrations were scored.

### 3.8. In Vitro Micronucleus Assay

Caco-2 cells (1.5 × 10^5^ cells/2 mL) were exposed to TiO_2_ (292 μg/mL) for 24 h without S9 mix (positive control: 25 μg/mL MMS) or treated with particles for 4 h followed by a recovery time of 20 h in the presence of S9 mix (positive control: 10 μg/mL CPA). After removing the supernatant, the cells were treated with 4.5 μg/mL of cytochalasin B for 30 h to prevent cytoplasmic division, but not nuclear division. After washing with PBS, the cells in complete medium were recovered for 1.5 h and then harvested. The cell pellets were re-suspended in 1 mL of 0.075 M KCl at 37 °C for 2 min to induce a rapid hypotonic shock, followed by fixation with methanol–acetic acid (3:1) for 5 min and 15 min and centrifugation (135× *g*, 5 min). The cells were then re-suspended in 1 mL of fixative solution, and 100 μL of the cell suspension was spread onto ice-cold slides and air-dried. Finally, the slides were stained with 5% Giemsa solution for 20 min. At least 200 well-spread cells were examined under a microscope (Axioskop 2 plus, Carl Zeiss), and the numbers of mononucleate, binucleate, and multinucleate cells were scored.

### 3.9. Statistical Analysis

All data were expressed as mean ± standard deviation. Statistical comparisons among groups were performed using one-way analysis of variance followed by Tukey’s test in SAS software version 9.4 (SAS Institute, Cary, NC, USA). A *p*-value of less than 0.05 was considered statistically significant.

## 4. Conclusions

The genotoxic potential of commercial food additive TiO_2_ particles of two different sizes (T3 and T4) was evaluated, focusing on DNA and chromosomal damage using in vitro diverse human intestinal cell systems. The results demonstrate that two different-sized TiO_2_ particles induced DNA fragmentations in Caco-2 and HT-29 cells as well as in Caco-2 monolayer, Caco-2/HT-29 co-culture, and FAE models at actual intake levels, regardless of metabolic activation. The DNA damage was highly correlated with oxidative stress caused by TiO_2_ particles, as evidenced by elevated ROS, antioxidant enzyme activity, and 8-OHdG levels. However, TiO_2_ particles did not induce in vitro chromosomal aberration or micronucleus formation. It is likely that TiO_2_ particles have the potential to induce oxidative stress-related DNA fragmentations; however, the extent of DNA damage does not appear to result in genotoxic effects at macroscopic levels, such as chromosomal aberrations and micronucleus formation. The extremely low oral absorption of food additive TiO_2_ particles and the potential protective role of food matrices on their genotoxicity should also be considered. Further in vivo studies are required to draw a definitive conclusion regarding the potential genotoxicity of food-grade TiO_2_ particles.

## Figures and Tables

**Table 1:** Average constituent particle sizes and size distributions of food-grade TiO_2_ particles (T3 and T4) measured from SEM images.

Sample	Average Size (nm)	Distribution (Number %)		
		<100 nm	100–200 nm	>200 nm
T3	161.1 ± 28.0	ND	89	11
T4	123.0 ± 26.7	18	82	ND

No significant differences were found between food-grade TiO_2_ particles (T3 and T4) (*p* > 0.05). Abbreviations: ND, not detectable.

**Table 2:** Frequency of chromosome aberrations in Caco-2 cells exposed to TiO_2_ particles (T3 and T4).

Treatment	Conc. (μg/mL)	S9 Mix	No. of Structural Aberrations					Gap	Total Aberration Cells (Excl Gaps)	Total Aberration Frequency (%)
			Chromatid Type		Chromosome Type		Fragmentation			
			Break	Exchange	Break	Exchange				
Control	0	−	1	0	0	1	2	0	4	2.0
Positive control (MMC)	0.2		12	0	1	4	14	1	31	15.5
T3	292		3	0	0	2	0	0	5	2.5
T4	292		3	0	0	1	0	1	4	2.0
Control	0	+	3	0	0	0	2	1	5	2.5
Positive control (CPA)	10		8	0	0	2	23	2	33	16.5
T3	292		5	0	0	1	0	2	6	3.0
T4	292		1	0	0	2	3	2	6	3.0

Abbreviations: Excl, excluding; MMC, mitomycin C; CPA, cyclophosphamide; −, without; +, with.

**Table 3:** Frequency of micronucleated binucleated cells (MNBNs) in Caco-2 cells exposed to TiO_2_ particles (T3 and T4).

Treatment	Conc. (μg/mL)	S9 Mix	MNBN (%)
Control	0	−	3.0
Positive control (MMS)	25		8.5
T3	292		2.5
T4	292		2.5
Control	0	+	2.5
Positive control (CPA)	10		9.0
T3	292		2.5
T4	292		3.0

Abbreviations: MMS, methyl methanesulfonate; CPA, cyclophosphamide; −, without; +, with.

## Data Availability

The original contributions presented in this study are included in the article. Further inquiries can be directed to the corresponding author(s).

## Abbreviations

The following abbreviations are used in this manuscript.

CAT	Catalase
CPA	Cyclophosphamide
DCF	Dichlorofluorescein
DDW	Distilled and deionized water
EFSA	European Food Safety Authority
EU	European Union
Excl	Excluding
FAE	Follicle-associated epithelium
FAO	Food and Agriculture Organization
FBS	Fetal Bovine Serum
FDA	United States Food and Drug Administration
8-OHdG	8-Hydroxy-2′-Deoxyguanosine
JECFA	Joint Expert Committee on Food Additives
LDH	Lactate dehydrogenase
MMC	Mitomycin C
MMS	Methyl methanesulfonate
MNBN	Micronucleated binucleated cell
ND	Not detectable
NP	Nanoparticle
PBS	Phosphate-buffered saline
ROS	Reactive oxygen species
SEM	Scanning electron microscopy
SOD	Superoxide dismutase
TEER	Transepithelial electrical resistance
WHO	World Health Organization
